# Beyond Hypertension: 24hr Blood Pressure Variability and Cognition

**DOI:** 10.1002/alz70860_104115

**Published:** 2025-12-23

**Authors:** Madeline Gibson, Katherine H. Franks, Ella Rowsthorn, Marina G. Cavuoto, Ian H Harding, Trevor T.‐J. Chong, Terence J O'Brien, Lucy Vivash, Stephanie Yiallourou, Matthew P. Pase

**Affiliations:** ^1^ Monash University, Melbourne, VIC, Australia; ^2^ Turner Institute for Brain and Mental Health, Monash University, Melbourne, VIC, Australia; ^3^ Turner Institute for Brain and Mental Health & School of Psychological Sciences, Monash University, Clayton, VIC, Australia; ^4^ School of Psychological Sciences & Turner Institute for Brain and Mental Health, Monash University, Melbourne, VIC, Australia; ^5^ Central Clinical School, Monash University, Melbourne, VIC, Australia; ^6^ Monash University, Clayton, VIC, Australia

## Abstract

**Background:**

Midlife hypertension is a well‐established risk factor for dementia. However, this knowledge is derived from research that uses office blood pressure (BP) measurements, single time‐point readings that fail to capture intraday BP fluctuations. Greater BP variability (fluctuations around an individual's average BP) may place additional strain on the vascular system, contributing to long‐term heart and brain health consequences.

**Methods:**

The sample comprised participants in a community‐based Brain and Cognitive Health (BACH) cohort, who completed cross‐sectional BP monitoring and neuropsychological assessments. Outcomes included a global composite cognitive score derived from principal component analysis and tests assessing key domains (WMS‐IV Visual Reproduction and Logical Memory, Trail Making Test [TMT], WAIS‐IV Similarities). BP variables included office, mean 12‐hour and 24‐hour BP, and BP variability calculated as the coefficient of variation (standard deviation divided by the mean) of 24‐hour systolic BP. Multiple linear regression assessed relationships between each BP variable and outcomes, adjusting for age, sex, education, body mass index, diabetic status, and anti‐hypertensive medication use.

**Results:**

The sample included 195 participants (aged 55–79 years, 55% women). Greater BP variability was associated with lower global cognitive scores (β=‐0.273 [95% CI: ‐0.467 to ‐0.078], *p* = .006). Higher BP variability was also associated with poorer executive function (TMT B‐A: β=2.190 [95% CI: 0.563 to 3.816]; TMT‐B: β=2.578, [95% CI: 0.831 to 4.324] *p* = .004; Similarities: β=‐0.224 [95% CI: ‐0.438 to ‐0.010], *p* = .041) and visual memory (immediate recall: β=‐0.363 [95% CI: ‐0.599 to ‐0.127], *p* = .003; delayed recall: β=‐0.592 [95% CI: ‐1.013 to ‐0.171]**,** *p* = .006) but not verbal memory. Results were unchanged in a sensitivity analysis that included further adjustments for office or mean 24hr BP. No other BP variables were significantly related to cognition.

**Conclusion:**

Higher BP variability was an independent predictor of poorer cognition. BP variability may be an important measure for prediction of cognitive health beyond office BP. Future research is needed to investigate whether BP variability is associated with dementia risk.

